# The Role of Prognostic Nutrition Index in Predicting Hospitalization of Patients With Heart Failure With Mildly Reduced Ejection Fraction

**DOI:** 10.7759/cureus.33102

**Published:** 2022-12-29

**Authors:** Ferhat S Yurdam, Yusuf Demir

**Affiliations:** 1 Department of Cardiology, Bakırçay University Çiğli Training and Research Hospital, İzmir, TUR

**Keywords:** hospitalization, prediction, prognostic nutritional index, heart failure, mildly reduced ejection fraction

## Abstract

Background

The prognostic nutritional index (PNI), consisting of albumin and lymphocyte counts, has been associated with satisfactory predictive values ​​for in-hospital mortality or clinical follow-up outcomes in acute situations. In this study, we aimed to evaluate the use of PNI for the prediction of hospital admission in individuals with mildly reduced ejection fraction heart failure (HFmrEF).

Methodology

This retrospective study was conducted between January 2019 and May 2022 and included 200 patients with HFmrEF detected by transthoracic echocardiography. Data from hospitalized patients (group 1) and outpatients (group 2) were compared.

Results

In the multivariable regression analysis, brain natriuretic peptide (odds ratio (OR) = 1.001; 95% confidence interval (CI) = 1.000-1.001, p = 0.001) and PNI (OR = 0.783; 95% CI = 0.720-0.853; p < 0.001) were independent predictors of hospital admission in patients with HFmrEF. The PNI value was statistically significantly higher in group 2 (52.36 ± 5.36) than in group 1 (38.3 ± 8.63, p < 0.001). The PNI value <46.75 is a predictor of hospitalization in patients with HFmrEF, with 86% sensitivity and 88% specificity.

Conclusions

Lower PNI levels predict hospital admission in HFmrEF patients. This measure, which can be easily evaluated in daily cardiological practice, allows for quick and precise decisions for hospitalization.

## Introduction

Chronic heart failure is a phenomenon associated with various risk factors, and its prevalence is increasing rapidly (2-3% globally) despite improvements in medical therapy. In the development of heart failure, nutrition-related dyslipidemia such as diabetes, obesity, and hypertension play a significant role [1]. One of the conditions linked to poor prognosis in hospitalized patients is malnutrition, affecting mainly the elderly [2]. Several chronic diseases, including heart failure, kidney failure, and cancer, may progress more rapidly in hospitalized malnourished individuals [3]. Attempts have been made to measure nutritional status using certain indices to predict prognosis [4-7]. Cardiac cachexia is a state of catabolic depletion linked with inflammation and neurohormonal activation, and it is thought to contribute to disease progression [8]. Cardiac cachexia is generally observed in those with advanced stages of chronic heart failure. Therefore, it is critical to monitor the nutritional state of heart failure patients [9].

There are numerous evaluation indices to determine nutritional status. These include the controlling nutritional status (CONUT), prognostic nutrition index (PNI), and geriatric nutrition risk index (GNRI) [10]. Biochemical markers and body mass index (BMI) are adequate for the calculation of these indexes. Because several nutritional indices, such as Mini-Nutritional Assessment-Short Form (MNA-SF) and Nutritional Risk Screening (NRS2002), are complex and the results may be influenced by the individuals evaluating the tests, it has become necessary to utilize more objective tests [11,12].

The PNI is a straightforward and objective metric that is derived from the total lymphocyte count and serum albumin levels. For individuals with acute heart failure, albumin and lymphocyte concentrations can provide crucial and independent information regarding prognosis. Albumin is affected by nutrition, liver, and kidney metabolism, with a negative impact on the severity of heart failure. Low lymphocyte count, which is one of the immunological activation indicators of the inflammatory system, has a negative impact on the outcomes of patients with heart failure and may signal less immune sensitivity and increased cortisol production [13].

Some studies have suggested that increases in inflammation and neurohormonal activation lead to worsening prognosis in cardiovascular diseases. In this study, we aimed to determine the value of PNI in predicting hospital admissions of individuals with mildly reduced ejection fraction heart failure (HFmrEF).

## Materials and methods

Study design and inclusion and exclusion criteria

This retrospective study included 200 patients who were diagnosed with HFmrEF between January 2019 and May 2022. In transthoracic echocardiography, heart failure patients with left ventricular ejection fraction (LVEF) of 40-50% were defined as HFmrEF. Patients over 18 years of age with HFmrEF were included in the study. Patients aged less than 18 years or more than 90 years, those suffering from acute coronary syndrome, and those with elevated acute inflammatory parameters (such as C-reactive protein (CRP) of >5 mg/L, procalcitonin >0.05 ng/mL, etc.) or liver function tests (aspartate aminotransferase (AST) and alanine aminotransferase (ALT) of >33 IU/L, prothrombin time (PT) of >14 seconds) were excluded from the study. The following data were recorded and analyzed: demographic data, comorbidities, heart failure information (etiology and functional capability according to the New York Heart Association (NYHA)) (Table 1), physical examination (e.g., blood pressure), blood test results, transthoracic echocardiography findings, and current medical therapy.

**Table 1:** NYHA classification (according to the American Heart Association guidelines). NYHA = New York Heart Association

Class	Patient symptoms
I	No limitation of physical activity. Ordinary physical activity does not cause undue fatigue, palpitation, and dyspnea (shortness of breath)
II	Slight limitation of physical activity. Comfortable at rest. Ordinary physical activity results in fatigue, palpitation, and dyspnea (shortness of breath)
III	Marked limitation of physical activity. Comfortable at rest. Less than ordinary activity causes fatigue, palpitation, or dyspnea
IV	Unable to carry on any physical activity without discomfort. Symptoms of heart failure at rest. If any physical activity is undertaken, discomfort increases

The patients were divided into two groups, namely, group 1 with hospitalized patients and group 2 with outpatients. Electrocardiogram data (heart rate and rhythm) of patients in group 1 were recorded during their emergency room evaluation prior to admission, whereas electrocardiogram data of patients in group 2 were retrieved from the hospital records.

Laboratory data definition

All biochemical analyses were conducted utilizing conventional procedures. Brain natriuretic peptide (BNP) was measured using the electrochemiluminescence method (Roche Diagnostic Indianapolis Elecsys 1010 auto-analyzer), and BNP >100 pg/mL was accepted as an indicator of cardiac dysfunction [14]. Patients’ routine hematological and biochemical blood tests were retrieved from hospital records. Chronic renal failure was defined as an estimated glomerular filtration rate (eGFR) of less than 60 mL/minute. PNI was computed using the following formula [15]: (10 × serum albumin in g/dL) + (0.005 × blood lymphocyte count/mm^3^).

Statistical analysis

Data analysis was conducted using SPSS for Windows version 15 (SPSS Inc., Chicago, IL, USA). The Kolmogorov-Smirnov test was used to assess the normality of the distribution of continuous variables. Normality of distribution analysis was performed for continuous data. Data with normal distribution were presented as mean and standard deviation, and data without normal distribution were presented as the interquartile range (IQR) (Q1-Q3). The number and frequency of categorical variables were reported using the Pearson chi-square test and Fisher’s exact test. Logistic regression was used to identify independent determinants of hospitalization among HFmrEF patients. Receiving operating characteristics (ROC) curve analysis was utilized to establish the optimal cut-off values to predict hospital admission in patients with HFmrEF. P-values less than 0.05 were considered statistically significant.

## Results

A total of 200 patients with HFmrEF with an average age of 66.2 ± 12.5 years (65% male, 35% female) were included in the study. Group 1 consisted of 50 (average age 74.5 ± 9.9 years, 50% male) and group 2 of 150 patients (average age 63.4 ± 12 years, 70% male). Individuals in group 1 were substantially older (p < 0.001), and the number of males was significantly higher in group 2 (p = 0.008).

Hypertension, diabetes mellitus, hyperlipidemia, and ischemic heart disease were seen in 47% (n = 94), 43% (n = 86), 75% (n = 150), and 74% (n = 148) of patients, respectively.

When clinical characteristics were compared, the presence of chronic renal failure was significantly different between groups (38% vs. 16%, p = 0.001, respectively). According to the functional capacity classification of NYHA, we found a statistically significant difference between groups 1 and 2 (p < 0.001). When functional capacity according to the NYHA classification was evaluated, in group 1, 2% were class 2, 62% were class 3, and 36% were class 4, whereas in group 2, 75% were class 1, and 25% were class 2. The rate of mortality was 16% (n = 8) in group 1 while no mortality was observed in group 2 (p < 0.001). Demographic data, comorbidities, and clinical features of patients are presented in Table 2.

**Table 2:** Demographic data, comorbid diseases, and clinical features. n = number of patients; SD = standard deviation; CRF = chronic renal failure; CVD = cerebrovascular diseases; PAD = peripheral arterial disease; CAD = coronary artery disease; COPD = chronic obstructive pulmonary disease; NYHA = New York Heart Association; Group 1 = patients with HFmrEF treated by hospitalization; Group 2 = outpatients with HFmrEF

Variables	Group 1 (n = 50)	Group 2 (n = 150)	P-value
Age, years, mean ± SD	74.5 ± 9.95	63.4 ± 12.09	<0.001
Male gender, n (%)	25 (50)	106 (70)	0.008
Hypertension, n (%)	20 (40)	74 (49)	0.25
Diabetes mellitus, n (%)	19 (38)	67 (44)	0.41
Hyperlipidemia, n (%)	32 (64)	108 (72)	0.29
CRF, n (%)	19 (38)	24 (16)	0.002
Smoke, n (%)	3 (6)	13 (8)	0.76
CVD history, n (%)	2 (4)	2 (1)	0.26
PAD, n (%)	3 (6)	9 (6)	1
COPD, n (%)	8 (16)	13 (8)	0.18
Thyroid disease, n (%)	4 (8)	19 (12)	0.45
Ischemic etiology, n (%)	39 (78)	109 (73)	0.57
Functional capacity, n (%)			
NYHA I	0	113 (75)	<0.001
NYHA II	1 (2)	37 (25)	
NYHA III	31 (62)	0	
NYHA IV	18 (36)	0	
Mortality, n (%)	8 (16)	0	<0.001

When electrocardiogram and echocardiographic findings were compared, only heart rate on electrocardiogram and systolic pulmonary arterial pressure (sPAP) on echocardiography were found to differ between groups. Heart rate (93.3 ± 17.9 vs. 76 ± 13 beats per minute, p < 0.001) and sPAP (33 (25-40) mmHg vs. 21.5 (18-30) mmHg, p < 0.001) were increased in group 1 when compared to group 2. There was no statistical difference between the two groups in LVEF values (45.47 ± 2.2% vs. 45.95 ± 2.7%, p = 0.26, respectively). Electrocardiography, echocardiography, and blood pressure data of patients are shown in Table 3.

**Table 3:** ECG, echocardiography, and blood pressure findings of the patients. Those with normal distribution of continuous variables are presented as the mean ± SD using Student’s t-test. Continuous variables that did not show normal distribution are presented as median and IQR (Q1-Q3) by applying the Mann-Whitney U test. ECG = electrocardiogram; n = number of patients; SD = standard deviation; LVEF = left ventricle ejection fraction; LVH = left ventricular hypertrophia; LVDD = left ventricular diastolic dysfunction; LVEDD = left ventricular end-diastolic diameter; LVESD = left ventricular end-systolic diameter; LA = left atrium; sPAP = systolic pulmonary arterial pressure; Group 1 = patients with HFmrEF treated by hospitalization; Group 2 = outpatients with HFmrEF

Variables	Group 1 (n = 50)	Group 2 (n = 150)	P-value
Electrocardiogram findings			
Heart rate, beats per minute, mean±SD	93.3 ± 17.9	76 ± 13	<0.001
Rhythm			
Sinus, n (%)	37 (74)	126 (84)	0.14
Atrial fibrillation, n (%)	13 (26)	24 (16)	
Blood pressure, mmHg			
Systolic, mean ± SD	128.2 ± 21.1	125.6 ± 15.5	0.36
Diastolic, median (IQR (Q1-Q3))	72.5 (65-85)	70 (65-85)	0.37
Echocardiographic findings			
LVEF, %, mean±SD	45.47 ± 2.2	45.95±2.7	0.26
LVH, n (%)	15 (30)	40 (26)	0.71
LVDD, n (%)	16 (32)	64 (42)	0.18
LVEDD, mm, mean ± SD	48.7 ± 4.8	49.1 ± 5.5	0.59
LVESD, mm, mean ± SD	31.7 ± 5.1	32.9 ± 5.7	0.19
LA diameter, mm, mean ± SD	41.2 ± 5.3	40.2 ± 5.9	0.30
sPAP, mmHg, median (IQR (Q1-Q3)	33 (25-40)	21.5 (18-30)	<0.001
Serious valve disease, n (%)			
Mitral regurgitation	2 (4)	5 (3)	1
Mitral stenosis	0	1 (0.6)	1
Aortic stenosis	1 (2)	4 (2)	1

At hospital presentation, patients were found to be using renin-angiotensin-aldosterone system (RAAS) inhibitors in 59.5% (n = 119), beta-blockers in 79.5% (n = 159), and diuretics in 72.5% (n = 145). Fifty-eight patients used at least one antithrombotic agent (Aspirin or clopidogrel). Table 4 presents the medical therapy of the patients.

**Table 4:** Drugs used by patients. n = number of patients; RAAS = renin-angiotensin-aldosterone system; Dhp CCBs = dihydropyridine calcium channel blockers; PTU = propylthiouracil; Group 1 = patients with HFmrEF treated by hospitalization; Group 2 = outpatients with HFmrEF

Prescribed drugs, n (%)	Group 1 (n = 50)	Group 2 (n = 150)	P-value
RAAS inhibitors	29 (58)	90 (60)	0.86
Beta-blockers	38 (76)	121 (80)	0.54
Aspirin	28 (56)	91 (60)	0.62
Clopidogrel	17 (34)	41 (27)	0.37
Anticoagulants	15 (30)	48 (32)	0.86
Statins	20 (40)	81 (54)	0.10
Dhp CCBs	12 (24)	26 (17)	0.30
Oral nitrate	6 (12)	12 (8)	0.39
Digoxin	1 (2)	6 (4)	0.50
Ivabradine	1 (2)	7 (4)	0.40
Diuretics			
Thiazide	13 (26)	54 (36)	0.19
Loop	18 (36)	34 (22)	0.06
Aldosterone antagonist	4 (8)	22 (14)	0.22
Oral antidiabetics	13 (26)	53 (35)	0.22
Insulin	10 (20)	25 (16)	0.67
Levothyroxine	3 (6)	11 (7)	1
Antithyroidal (PTU, methimazole)	1 (2)	1 (0.7)	0.44

When hematological and biochemical blood parameters were analyzed, neutrophil levels (6.64 ± 2.47 × 10^3^/L vs. 4.96 ± 1.64 × 10^3^/L, p < 0.001), creatinine levels (1.38 ± 0.98 mg/dL vs. 0.94 ± 0.32 mg/dL, p < 0.001), and BNP levels (1,199 (646-3,577) pg/mL vs. 195 (146-333) pg/mL, p < 0.001) were significantly increased in group 1 when compared to group 2. On the other hand, lymphocyte (1.42 ± 0.76 × 10^3^/L vs. 2.1 ± 0.77 × 10^3^/L, p < 0.001), albumin level (3.1 ± 0.62 mg/dL vs. 4.2 ± 0.42 mg/dL, p < 0.001), and PNI (38.3 ± 8.63 vs. 52.6 ± 5.36, p < 0.001) were significantly higher in group 2 when compared to group 1. Hematological and biochemical blood values ​​are shown in detail in Table 5.

**Table 5:** Hematological and biochemical laboratory values of the patients. Those with the normal distribution of continuous variables are presented as the mean ± SD using Student’s t-test. Continuous variables that did not show normal distribution were presented as median and IQR (Q1-Q3) by applying the Mann-Whitney U test. n = number of patients; WBC = white blood cell; Neu = neutrophil; Lym = lymphocyte; Hb = hemoglobin; Plt = platelet; PNI = prognostic nutritional index; TG = triglyceride; HDL = high-density lipoprotein; LDL = low-density lipoprotein; BNP = brain natriuretic peptide; TSH = thyroid-stimulating hormone; Group 1 = patients with HFmrEF treated by hospitalization; Group 2 = outpatients with HFmrEF

Variables	Group 1 (n = 50)	Group 2 (n = 150)	P-value
WBC count, ×10^3^/L, median (IQR (Q1-Q3))	8.40 (6.75-9.62)	7.85 (6.50-9.37)	0.22
Neu count, ×10^3^/L, median (IQR (Q1-Q3))	6.60 (4.82-7.80)	4.90 (3.80-5.97)	<0.001>
Lym count, ×10^3^/L, median (IQR (Q1-Q3))	1.40 (0.90-1.80)	1.90 (1.60-2.50)	<0.001>
Hb, g/dL, mean ± SD	12.99 ± 1.24	13.23 ± 1.52	0.32
Plt count, ×10^3^ /L, median (IQR (Q1-Q3))	254 (203-290)	236 (197-266)	0.25
Glucose, mg/dL, median (IQR (Q1-Q3))	118 (97-147)	108 (97-137)	0.52
Creatinine, mg/dL, mean ± SD	1.38 ± 0.98	0.94 ± 0.32	<0.001>
Sodium, mEq/L, mean ± SD	138 ± 4.2	138 ± 2.5	0.55
Potassium, mEq/L, mean ± SD	4.3 ± 0.49	4.4 ± 0.43	0.32
Albumin, mg/dL, mean ± SD	3.1 ± 0.62	4.2 ± 0.42	<0.001>
Uric acid, mean ± SD	5.8 ± 2.1	5.7 ± 1.9	0.36
PNI, mean ± SD	38.3 ± 8.63	52.6 ± 5.36	<0.001>
Total cholesterol, mg/dL, median (IQR (Q1-Q3))	161 (129-186)	170 (145-200)	0.051
TG, mg/dL, median (IQR (Q1-Q3))	114 (85-169)	131 (101-179)	0.06
HDL, mg/dL, median (IQR (Q1-Q3))	38 (32-45)	41 (35-49)	0.11
LDL, mg/dL, median (IQR (Q1-Q3))	92 (67-111)	95 (72-126)	0.34
BNP, pg/mL, median (IQR (Q1-Q3))	1,199 (646-3,577)	195 (146-333)	<0.001>
TSH, mIU/L, mean ± SD	1.69 ± 1.34	1.91 ± 1.56	0.38

In the univariable regression analysis of the factors affecting the hospitalization in HFmrEF patients, age, sex, CRF, PNI, sPAP, heart rate, neutrophil, and BNP were predictors. Multivariable logistic regression analysis revealed that high heart rate (odds ratio (OR) = 1.069; 95% confidence interval (CI) 1.024-1.115, p = 0.002), high BNP (OR = 1.001; 95% CI = 1.000-1.001, p = 0.001), and low PNI (OR = 0.783; 95% CI = 0.720-0.853, p < 0.001) were independent predictors of hospital admission in patients with HFmrEF (Table 6).

**Table 6:** Univariate and multivariate logistic regression analyses in predicting hospitalization in patients with HFmrEF. OR = odds ratio; CI = confidence interval; CRF = chronic renal failure; PNI = prognostic nutritional index; BNP = brain natriuretic peptide; sPAP = systolic pulmonary arterial pressure; HFmrEF = mildly reduced ejection fraction heart failure

	Univarite logistic regression			Multivariate logistic regression		
Variables	OR	95% CI	P-value	OR	95% Cl	P-value
Age	1.096	1.057-1.135	<0.001	1.009	0.953-1.068	0.75
Male gender	2.409	1.250-4.644	0.009	0.977	0.262-3.639	0.97
CRF	0.311	0.151-0.638	0.001	1.732	0.480-6.254	0.40
PNI	0.746	0.687-0.811	<0.001	0.788	0.716-0.867	<0.001
BNP	1.001	1.000-1.001	<0.001	1.001	1.000-1.001	0.004
sPAP	1.083	1.046-1.112	<0.001	1.001	0.931-1.076	0.97
Heart rate	1.076	1.048-1.103	<0.001	1.069	1.024-1.115	0.002
Neutrophil	1.000	1.000-1.001	<0.001	1.000	1.000-1.001	0.13

**Figure 1:**
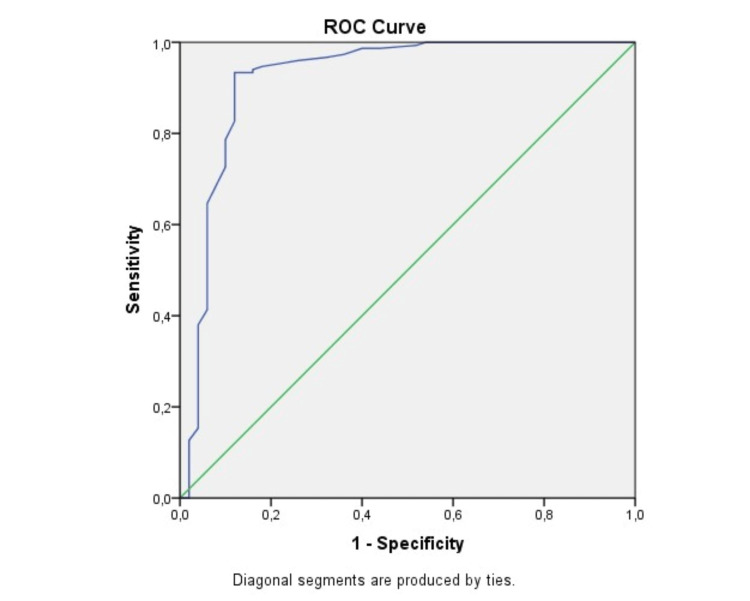
Analysis of optimal cut-off values of the ROC curve PNI distribution. ROC = receiver operating characteristic; PNI = prognostic nutritional index

ROC curve analysis revealed that PNI <46.75 had a 86% sensitivity and 88% specificity for the prediction of hospital admission (AUC = 0.921, 95% CI = 0.864-0.979, p < 0.001) (Figure 1).

## Discussion

PNI, an indication of nutritional status, was independently demonstrated to predict hospital admission in those with HFmrEF in this study.

When assessing nutritional status, it is preferable to utilize a metric with a strong predictive value, such as the PNI, rather than albumin or lymphocyte counts alone. In a retrospective cohort of 1,673 patients, Cheng et al. reported that nutritional status, as measured by PNI, is independently associated with both short and long-term total and cardiovascular mortality in patients with acute heart failure [16].

The pathophysiology of malnutrition in heart failure is due to a decrease in blood flow in the gastrointestinal system that leads to decreased intestinal blood flow, increased inflammation, local edema, and increased tissue permeability.

In our study, patients in the group with high sPAP had lower PNI and more severe malnutrition. These findings correlate with the findings of Anker et al. and Valentova et al. Anker et al. [17] reported that left ventricular systolic functions were nearly similar between those with heart failure that had severe malnutrition and less severe malnutrition. The relationship between dietary status and left ventricular function is controversial, with inconsistent findings in the literature. However, studies that used the nutritional risk index (NRI) to evaluate the nutrition state of heart failure patients with systolic ventricular dysfunction found no connection between dietary status and left ventricular function [18]. Valentova et al. [19] reported that intestinal edema is induced by an increase in right ventricular pressure (increased sPAP), which ultimately results in cardiac cachexia; however, this is not related to left ventricular dysfunction. Kinugasa et al. and Liu et al. [20] have previously demonstrated that nutritional status was not related to prognosis in patients with heart failure in which ejection fraction was preserved or low. The mechanisms in the pathophysiology of congestion in cardiac cachexia are thought to be a combination of starvation and inflammation in both heart failure with low or preserved ejection fraction [21].

When PNI was measured at the baseline, lower PNI values were reported to predict a poorer outcome in 200 individuals who had stomach cancer surgery [16]. Sun et al. [22] confirmed the usefulness of PNI for detecting survival and complication rates in various forms of cancer. In individuals with acute heart failure, PNI is reported to correlate with survival [23]. A previous study reported that decreased albumin was a predictor of shorter survival, and lower lymphocyte concentrations were linked to greater mortality in patients with advanced heart failure [24].

In a prospective study of individuals with preserved ejection fraction heart failure, Agus et al. [25] found that PNI values less than 37 could predict increased overall mortality and inpatient re-admission. The strong correlation between PNI components and multiple concomitant diseases may be the reason why higher PNI values were reported previously in ambulatory and clinically stable individuals with heart failure [24]. Tekin et al. [26] recently reported that those with low PNI were at an increased risk of cardiovascular mortality or hospital admission due to worsening heart failure and persistent left ventricular systolic dysfunction when predicting prognosis in patients with peripartum cardiomyopathy. In a study of 110 patients with chronic renal failure who had hemodialysis and underwent heart surgery, Kurumisawa and Kawahito reported that patients with low PNI had considerably greater rates of in-hospital mortality, complications, and postoperative hospital stays [27]. In an evaluation of kidney donors, Hori et al. [28] reported that one year after donor nephrectomy decreased PNI was associated with lower renal function postoperatively. In addition, a prior investigation that evaluated the effect of PNI on cardiovascular disease-related mortality in those undergoing peritoneal dialysis demonstrated that PNI was a stronger predictor of cardiovascular disease mortality when compared to hemoglobin and leukocytes [29]. A similar relationship has been reported in individuals who undergo continuous ambulatory peritoneal dialysis. We believe chronic renal failure plays a role because it influences hospitalization and worsens prognosis through a synergistic impact with heart failure and may lead to decompensation. However, chronic renal failure was less predictive of hospitalization in our study.

Sze et al. [30] studied the prevalence of malnutrition and its predictive significance in heart failure patients. Malnutrition was far more prevalent in individuals with high levels of N-terminal-pro-hormone BNP (>4,000 ng/L). This study demonstrated that BNP is a predictor of hospitalization.

Limitations

Due to the study’s single-center nature, its retrospective design, and the relatively low number of patients, our findings cannot be generalized. Further large-scale studies are required to confirm our findings.

## Conclusions

Low PNI levels are a marker of malnutrition and prognosis. This measure, which can easily and inexpensively be calculated in regular cardiology practice, may allow clinicians to make hospitalization decisions quickly and simply.
